# Cardiovascular Risk Factors Are Correlated with Low Cognitive Function among Older Adults Across Europe Based on The SHARE Database

**DOI:** 10.14336/AD.2017.0128

**Published:** 2018-02-01

**Authors:** Joana Lourenco, Antonio Serrano, Alice Santos-Silva, Marcos Gomes, Claudia Afonso, Paula Freitas, Constanca Paul, Elisio Costa

**Affiliations:** ^1^Family Health Unit of Pedras Rubras, Maia, Portugal; ^2^Faculty of Nutrition and Food Sciences, University of Porto, Porto, Portugal; ^3^Center for Health Technology and Services Research (CINTESIS), Porto, Portugal; ^4^UCIBIO, REQUIMTE and Faculty of Pharmacy, University of Porto, Porto, Portugal; ^5^Department of Endocrinology, Diabetes and Metabolism, Centro Hospitalar São João, Porto, Portugal; ^6^Faculty of Medicine, University of Porto, Porto, Portugal; ^7^Institute of Biomedical Sciences Abel Salazar (ICBAS), University of Porto, Porto, Portugal

**Keywords:** cognitive function, ageing, chronic diseases, cardiovascular risk factors

## Abstract

Increased life expectancy is associated with a high prevalence of chronic, non-communicable diseases including cognitive decline and dementia. The purpose of this study was to evaluate the prevalence of cognitive impairment using three cognitive abilities (verbal fluency, numeracy and perceived memory) and their association with cardiovascular risk factors in seniors across Europe. Data from participants in wave 4 of the SHARE (Survey of Health, Ageing, and Retirement in Europe) database was used. Cognitive performance in perceived memory, verbal fluency and numeracy was evaluated using simple tests and a memory complaints questionnaire. Clinical and sociodemographic variables were also studied for potential associations. Standardised prevalence rates of cognitive impairment based on age and gender were calculated by country. The prevalence of cognitive impairment was 28.02% for perceived memory, 27.89% for verbal fluency and 20.75% for numeracy throughout the 16 evaluated countries. Years of education, being a current or former smoker, number of chronic diseases, diabetes or hyperglycemia, heart attack and stroke were all independent variables associated with impairment in the three studied cognitive abilities. We also found independent associations between physical inactivity and verbal fluency and numeracy impairment, as well as hypertension and perceived memory impairment. Lower performance in the evaluated cognitive abilities and higher memory complaints are highly prevalent, have a heterogeneous distribution across Europe, and are associated with multiple factors, most of which are potentially preventable or treatable, especially cardiovascular risk factors.

Given worldwide population projections indicate that the number of persons aged 60 or above will double by 2050 and triple by 2100 [1]. Increased life expectancy is associated with an increased prevalence of chronic diseases, cognitive decline and dementia [2]. The cellular and molecular mechanisms associated with cognitive impairment are not well-established [3]; however, recent studies suggest that the pathological processes starts 10-20 years before the clinical onset of dementia [4]. The most commonly diagnosed cause of cognitive impairment is Alzheimer’s disease (AD), but vascular diseases including subclinical brain injury, stroke and silent brain infarction are also important causes and contributors to cognitive decline [5]. In fact, an association between brain health and cardiovascular risk factors was established in middle-aged adults using magnetic resonance imaging [6]. Another study also suggested a link between neurocognitive function and systemic vascular health through the evaluation of arterial stiffness and reactivity [7]. Moreover, silent strokes are more common than strokes with clinical manifestations and constitute an important risk factor as precursors of future strokes and cognitive impairment [3]. It is estimated that more than one in 10 adults over 60 years of age has had a stroke resulting in the loss of cognitive ability [8].

The impact of cardiovascular risk factors on cognitive function is receiving heightened attention as modifications of these factors may reduce the risk of dementia later in life. Therefore, we first aimed to evaluate the prevalence of low cognitive function by assessing perceived memory, verbal fluency and numeracy in people over 50 years of age across 16 European countries. Secondly, we assessed the association between cardiovascular risk factors and cognitive function for the three cognitive abilities listed above. The identification of factors associated with impaired cognitive abilities in older adults can be used to develop effective interventions that prevent declines in the cognitive function of seniors across Europe.

## MATERIAL AND METHODS

In this work, we used data from the SHARE (Survey of Health, Ageing, and Retirement in Europe) project, wave 4. SHARE is an international European database containing detailed information pertaining to the demographics, health, and social and economic status from representative samples of community-based populations over 50 years of age from 16 European countries (Austria, Belgium, France, Germany, Netherlands, Switzerland, the Czech Republic, Hungary, Poland, Denmark, Sweden, Estonia, Spain, Portugal, Italy, Slovenia) [9]. Modelled after the US Health and Retirement Study and the English Longitudinal Study of Ageing, SHARE contains data from a very large population. The harmonised, cross-national design allows for consistent international comparisons of a large number of factors simultaneously, providing a dynamic picture of ageing in Europe. A detailed description of the SHARE data and methodology has been published and is available to registered users on the SHARE website (http://www.share-project.org).

The wave from 2010 contains data from 58,489 individuals aged 23-103 years. To evaluate the prevalence of and the variables associated with poorer cognitive performance in three cognitive abilities (perceived memory, verbal fluency and numeracy), our sample included non-institutionalised individuals aged 50 years or more, with a body mass index (BMI) of 18.5 kg/m^2^ or higher, and who answered all of the questions included in this analysis (dependent and independent variables). We excluded data from three countries (Germany, Sweden and Poland) in our association analysis due to the low number of individuals that remained after exclusion of cases with missing values (less than 5%).

### Dependent variables - cognitive abilities

Two domains of cognitive function, i.e. verbal fluency (a test of executive function) and numeracy (arithmetical calculations) were evaluated in this work. The third cognitive domain, perceived memory, was assessed by a memory complaint questionnaire. In the SHARE project, cognitive tasks were based on standard dementia screening tools, i.e. the Mini-Mental-State and Dementia Detection Scale [10].

### Perceived memory

The participant’s memory complaints in SHARE were assessed through the questions “How would you rate your memory at the present time? Would you say it is ‘excellent’, ‘very good’, ‘good’, ‘fair’ or ‘poor’?” This variable was dichotomised as excellent, very good or good, and as fair or poor [11].

### Numeracy

The participant’s numeracy was assessed through the following described mathematical tasks. Participants were asked, “If the chance of getting a disease is 10 percent, how many people out of one thousand would be expected to get the disease?” The participant was then asked a second question: “In a sale, a shop is selling all items at half price. Before the sale, a sofa costs 300 (local currency). How much will it cost in the sale?” If the participant answered the first question incorrectly, the numeracy test was stopped after the second question, irrespective of whether the participant answered the second question correctly. If the first question was answered correctly, a third question was posed: “A second hand car dealer is selling a car for 6,000 (local currency). This is two-thirds of what it costs new. How much did the car cost new?” If two questions were answered correctly, the participant was invited to answer the last question: “Let’s say you have 2,000 (local currency) in a savings account. The account earns ten percent interest each year. How much would you have in the account at the end of two years?” The numeracy score ranged from 1 (the participant did not answer correctly any of the math questions) to 5 (the participant answered all of the math questions correctly). This variable was dichotomised into two categories, a score of 3 or less (indicative of impairment) or a score of more than 3 [12].

**Table 1:** Demographic characteristics of the sample population included in this study by gender and age group.

Country	Total SHARE Population (n)	Selected Population (n)	Selected Population (%)	Total Male (n)	Selected Male (n)	Selected Male (%)	Total Female (n)	Selected Female (n)	Selected Female (%)	50-54	Male (n) 55 - 64	65-74	>75 years	50-54	Female (n) 55-64	65-74	>75 years
Austria	5286	3953	74.78	2230	1706	76.5	3056	2247	73.53	345	607	517	237	382	843	679	343
Germany*	1572	16	1.02	736	6	0.82	836	10	1.2	2	2	0	2	1	7	1	1
Sweden*	1951	65	3.33	894	31	3.47	1057	34	3.22	4	8	14	5	4	13	10	7
Netherlands	2762	756	27.37	1220	342	28.03	1542	414	26.85	64	127	86	65	74	144	129	67
Spain	3570	1316	36.86	1606	622	38.73	1964	694	35.34	132	213	148	129	149	237	154	154
Italy	3583	1346	37.57	1605	613	38.19	1978	733	37.06	97	263	175	78	107	309	201	116
France	5857	3298	56.31	2512	1455	57.92	3345	1843	55.1	246	534	405	270	291	682	524	346
Denmark	2276	431	18.94	1036	210	20.27	1240	221	17.82	28	85	48	49	35	77	52	57
Switzerland	3750	2371	63.23	1682	1121	66.65	2068	1250	60.44	151	405	310	255	172	453	370	255
Belgium	5300	2695	50.85	2363	1237	52.35	2937	1458	49.64	77	431	445	284	75	551	524	308
Czech	6118	4310	70.45	2576	1843	71.55	3542	2467	69.65	240	635	519	449	338	814	688	627
Poland*	1724	42	2.44	753	20	2.66	971	22	2.27	0	8	9	3	4	4	7	7
Hungary	3076	2843	92.43	1322	1223	92.51	1754	1620	92.36	122	570	320	211	182	707	441	290
Portugal	2080	1606	77.21	895	711	79.44	1185	895	75.53	108	233	214	156	136	292	243	224
Slovenia	2756	2518	91.36	1196	1108	92.64	1560	1410	90.38	149	363	362	234	201	464	426	319
Estonia	6828	6014	88.08	2748	2392	87.05	4080	3622	88.77	499	807	640	446	740	1263	941	678

* These countries were not evaluated for an association between cardiovascular risk factors and cognitive function.

### Verbal fluency

This test measured executive function and language abilities. Participants were told: “Now, I would like you to name as many different animals as you can think of. You have one minute to do this. Ready, go.” Valid answers were any members of the animal kingdom, real or mythical, specific species’ names, any accompanying breeds within the species, as well as male, female, and infant names within the species. Proper nouns were considered incorrect. The verbal fluency score was the number of valid animal names the participant was able to state. This result was dichotomised into two categories: a score of 15 or less, suggestive of verbal fluency impairment, or a score of more than 15 [13].

### Explanatory variables

The wide scope of information in the SHARE project allowed us to include a large number of putative explanatory variables, such as sociodemographic (age, gender and years of education) and health variables.

**Table 2:** Standardised prevalence of impaired cognitive performance by country and gender.

	Perceived memory impairment			Numeracy impairment			Verbal fluency impairment		
	Overall prevalence (95% CI)	Standardised incidence rates (95% CI)		Overall prevalence (95% CI)	Standardised incidence rates (95% CI)		Overall prevalence (95% CI)	Standardised incidence rates (95% CI)	
		Male	Female		Male	Female		Male	Female
Austria	14.66 (14.40-14.94)	15.33 (14.94-15.72)	14.00 (14.63 -14.38)	12.60 (12.35-12.85)	9.61 (9.30-9.92)	15.60 (15.21- 15.99)	18.92 (18.62-19.23)	18.28 (17.86-18.71)	19.56 (19.12-20.01)
Germany*	13.55 (13.30 -13.81)	0.00 (0.00-0.01)	27.11 (26.59-27.63)	21.52 (21.20-21.85)	11.54 (11.20-11.88)	31.50 (30.95-32.06)	14.93 (14.66-15.20)	11.54 (11.20-11.88)	18.32 (17.89-18.74)
Sweden*	22.74 (22.40-23.07)	31.86 (31.30-32.42)	13.61 (13.25-13.98)	5.82 (5.65-5.99)	3.85 (3.65-4.05)	7.78 (7.51-8.07)	14.26 (14.00-14.53)	18.88 (18.45-19.31)	9.64 (9.34-9.96)
Netherlands	9.79 (9.58-10.02)	8.72 (8.43-9.02)	10.86 (10.54-11.20)	12.85 (12.60-13.10)	7.22 (6.96-7.50)	18.47 (18.05-18.91)	17.76 (17.46-18.05)	17.35 (16.94-17.77)	18.16 (17.74-18.59)
Spain	37.93 (37.50-38.37)	34.91 (34.32-35.50)	40.96 (40.33-41.60)	46.02 (45.55-46.50)	38.28 (37.67-38.90)	53.76 (53.04-54.49)	58.91 (58.37-59.45)	55.07 (54.34-55.81)	62.75 (61.97-63.54)
Italy	27.34 (26.98-27.71)	24.81 (24.32-25.31)	29.87 (29.33-30.42)	28.81 (28.44-29.19)	20.99 (20.54-21.45)	36.63 (36.03-37.24)	62.57 (62.01-63.12)	60.31 (59.54-61.08)	64.82 (64.03-65.63)
France	26.54 (26.18-26.91)	25.43 (24.93-25.93)	27.66 (27.14-28.19)	23.84 (23.50-24.19)	18.42 (18.00-18.85)	29.26 (28.73-29.80)	33.82 (33.42-34.23)	32.51 (31.95-33.08)	35.13 (34.55-35.72)
Denmark	8.82 (8.62-9.03)	7.05 (6.79-7.32)	10.60 (10.28-10.93)	10.89 (10.66-11.12)	7.19 (6.92-7.46)	14.59 (14.21-14.97)	6.48 (6.31-6.67)	6.67 (6.42-6.93)	6.30 (6.05-6.55)
Switzerland	12.85 (12.60-13.11)	13.75 (13.39-14.13)	11.95 (11.61-12.30)	9.04 (8.83-9.25)	6.67 (6.42-6.94)	11.41 (11.07-11.75)	22.91 (22.57-23.24)	24.25 (23.77-24.75)	21.56 (21.10-22.03)
Belgium	20.34 (20.03-20.66)	19.52 (19.09-19.97)	21.16 (20.70-21.62)	16.82 (16.54-17.11)	11.99 (11.65-12.34)	21.66 (21.20-22.12)	20.91 (20.59-21.23)	20.10 (19.66-20.55)	21.71 (21.25-22.18)
Czech	24.42 (24.08-24.77)	24.30 (23.81-24.79)	24.55 (24.06-25.05)	17.01 (16.72-17.30)	12.99 (12.64-13.35)	21.03 (20.58-21.49)	16.95 (16.66-17.24)	15.78 (15.39-16.18	18.11 (17.69-18.54)
Poland*	15.95 (15.67-16.23)	14.00 (13.63 -14.37)	17.90 (17.49-18.33)	29.99 (29.60-30.37)	31.36 (30.80-31.92)	28.62 (28.09-29.15)	41.20 (40.75-41.65)	46.37 (45.69-47.05)	36.03 (35.44-36.64)
Hungary	32.70 (32.30-33.11)	31.33 (30.78-31.90)	34.07 (33.49-34.65)	18.54 (18.24-18.84)	15.93 (15.54-16.33)	21.14 (20.69-21.61)	39.01 (38.57-39.45)	38.83 (38.21-39.45)	39.19 (38.57-39.81)
Portugal	44.41 (43.94-44.88)	38.35 (37.74-38.97)	50.46 (49.76-51.17)	36.92 (36.50-37.35)	26.89 (26.38-27.41)	46.95 (46.27-47.64)	63.11 (62.55-63.67)	59.65 (58.88-60.42)	66.57 (65.76-66.38)
Slovenia	25.38 (25.03-25.74)	23.89 (23.41-24.38)	26.88 (26.37-27.40)	29.83 (29.45-30.22)	25.05 (24.55-25.55)	34.62 (34.03-35.21)	24.07 (23.73-24.42)	23.74 (23.26-24.23)	24.40 (23.92-24.90)
Estonia	46.18 (45.71-46.66)	46.68 (46.00-47.36)	45.69 (45.02-46.37)	18.79 (18.48-19.09)	15.87 (15.48-16.27)	21.70 (21.24-22.17)	19.28 (18.97-19.59)	19.84 (19.40-20.28)	18.73 (18.30-19.16)

* Germany, Sweden and Poland have a small number of cases.

Age was calculated as the difference between the year 2010 and the date of birth, and four age classes were set (50-54, 55-64, 65-74 and more than 75 years old). The gender response generated a dichotomous variable, male or female. The education variable resulted from their response to the question “years of education”, which was dichotomised as ≤ 12 years or as > 12 years of education. The self-reported presence or absence of physician-diagnosed vascular diseases (“heart attack”, stroke, diabetes or hyperglcemia, and high blood pressure or hypertension) as well as smoking status (current or former smoker) were recorded. The continuous variable BMI was transformed into a discrete variable with three classes (18.5-24.9, 25-29.9 and 30 kg/m^2^ or higher). The variable “number of chronic diseases” was based on the number of chronic diseases reported by each individual, dichotomised by ≤ 2 or >2 chronic diseases. Physical inactivity was assessed on the basis of the following questions: “How often do you engage in activities that require a moderate level of energy such as gardening, cleaning the car, or going for a walk?” and “We would like to know about the type and amount of physical activity you do in your daily life. How often do you engage in vigorous physical activity, such as sports, heavy housework, or a job that involves physical labor?” These questions addressed their levels of moderate and vigorous physical activity, respectively. Physical inactivity was defined as never, or almost never, engaging in moderate or vigorous physical activity.

**Table 3:** Association of explanatory variables with memory impairment; unadjusted and adjusted models.

	n	n (%) of fair or poor	Unadjusted model			Adjusted model *		
	33126		OR	CI 95	p	OR	CI 95	p
Female	18709	5430 (29.0)	1	-	-	1	-	-
Male	14417	3867 (26.8)	0.896	0.854-0.941	<0.001	0.927	0.879-0.977	0.005
Age, years								
>75	6460	1951 (30.2)	1	-	-	1	-	-
65-74	9504	2658 (28.0)	0.897	0.837-0.962	0.002	0.903	0.840-0.970	0.005
55-64	12048	3225 (26.8)	0.845	0.790-0.903	<0.001	0.849	0.793-0.910	<0.001
50-54	5114	1463 (28.6)	0.926	0.854-1.004	0.062	0.936	0.861-1.018	0.121
Education, years								
>12-y	9376	1689 (18.0)	1	-	-	1	-	-
≤12-y	23750	7608 (32.0)	2.145	2.022-2.276	<0.001	1.968	1.852-2.091	<0.001
BMI, kg/m^2^								
≥30	7652	2386 (31.2)	1	-	-	-	-	-
25-29.9	13634	3808 (27.9)	0.855	0.805-0.909	<0.001	-	-	-
18.5-24.9	11840	3103 (26.2)	0.784	0.736-0.835	<0.001	-	-	-
Smoking								
Smoker*	18156	5247 (28.9)	1	-	-	1	-	-
No	14970	4050 (27.1)	0.912	0.869-0.958	<0.001	0.928	0.881-0.978	0.005
Physical inactivity								
No	29013	8116 (28.0)	1			-	-	-
Yes	4113	1181 (28.7)	0.964	0.897-1.036	0.323	-	-	-
Number of chronic diseases								
>2	9050	3819 (42.2)	1	-	-	1	-	-
≤2	24076	5478 (22.8)	0.403	0.383-0.425	<0.001	0.574	0.537-0.614	<0.001
Comorbidities								
Heart attack								
Yes	4805	2096 (43.6)	1	-	-	1	-	-
No	28321	7201 (25.4)	0.441	0.414-0.469	<0.001	0.653	0.609-0.701	<0.001
Stroke								
Yes	1485	804 (54.1)	1	-	-	1	-	-
No	31641	8493 (26.8)	0.311	0.280-0.345	<0.001	0.479	0.429-0.536	<0.001
Diabetes or high blood sugar								
Yes	4135	1583 (38.3)	1	-	-	1	-	-
No	28991	7714 (26.6)	0.584	0.546-0.626	<0.001	0.900	0.834-0.970	0.006
High blood pressure								
Yes	13241	4533 (34.2)	1	-	-	1	-	-
No	19885	4764 (24.0)	0.605	0.577-0.635	<0.001	0.877	0.829-0.928	<0.001

* Country random effect: estimate = 0.006; standard error = 0.040; p = 0.881.

### Statistical analysis

A descriptive analysis of the outcomes was performed in order to obtain an estimate of the proportion of individuals with cognitive impairment in the 16 European countries. Age and gender standardised prevalence of cognitive impairment, i.e. in perceived memory, numeracy and verbal fluency, was calculated by country and the 95% confidence intervals (95% CI) were calculated. We used direct age-adjusted standardisation based on the standard European population of 2013 [14].

With individuals nested by country, a multilevel logistic regression approach was used considering the three cognitive abilities as dependent variables: perceived memory (0: “poor” or “fair”; 1: “good”, “very good,” “excellent”), verbal fluency (0: ≤ 15 correct words; 1: > 15 correct words), and numeracy (0: “bad” 1-3, 1: “good” 4-5). In the first approach, multilevel, univariate, logistic regression models were used considering each covariate to identify potential factors associated with the outcome variables. Significant covariates retained in the first step (p < 0.05) were included in a multilevel, multivariable, logistic regression model. Country was considered to be a random effect. The final model was composed only of significant covariates, as determined by a backward selection method. Odds ratios and their 95% CI are reported. All the analyses were performed using the software IBM SPSS (version 23) and p < 0.05 was considered significant.

**Table 4:** Association of explanatory variables with verbal fluency impairment; unadjusted and adjusted models.

	n	n (%) of fair or poor	Unadjusted model			Adjusted model *		
	33126		OR	CI 95	p	OR	CI 95	p
Female	18709	5430 (29.0)	1	-	-	1	-	-
Male	14417	3867 (26.8)	0.896	0.854-0.941	<0.001	0.927	0.879-0.977	0.005
Age, years								
>75	6460	1951 (30.2)	1	-	-	1	-	-
65-74	9504	2658 (28.0)	0.897	0.837-0.962	0.002	0.903	0.840-0.970	0.005
55-64	12048	3225 (26.8)	0.845	0.790-0.903	<0.001	0.849	0.793-0.910	<0.001
50-54	5114	1463 (28.6)	0.926	0.854-1.004	0.062	0.936	0.861-1.018	0.121
Education, years								
>12-y	9376	1689 (18.0)	1	-	-	1	-	-
≤12-y	23750	7608 (32.0)	2.145	2.022-2.276	<0.001	1.968	1.852-2.091	<0.001
BMI, kg/m^2^								
≥30	7652	2386 (31.2)	1	-	-	-	-	-
25-29.9	13634	3808 (27.9)	0.855	0.805-0.909	<0.001	-	-	-
18.5-24.9	11840	3103 (26.2)	0.784	0.736-0.835	<0.001	-	-	-
Smoking								
Smoker*	18156	5247 (28.9)	1	-	-	1	-	-
No	14970	4050 (27.1)	0.912	0.869-0.958	<0.001	0.928	0.881-0.978	0.005
Physical inactivity								
No	29013	8116 (28.0)	1			-	-	-
Yes	4113	1181 (28.7)	0.964	0.897-1.036	0.323	-	-	-
Number of chronic diseases								
>2	9050	3819 (42.2)	1	-	-	1	-	-
≤2	24076	5478 (22.8)	0.403	0.383-0.425	<0.001	0.574	0.537-0.614	<0.001
Comorbidities								
Heart attack								
Yes	4805	2096 (43.6)	1	-	-	1	-	-
No	28321	7201 (25.4)	0.441	0.414-0.469	<0.001	0.653	0.609-0.701	<0.001
Stroke								
Yes	1485	804 (54.1)	1	-	-	1	-	-
No	31641	8493 (26.8)	0.311	0.280-0.345	<0.001	0.479	0.429-0.536	<0.001
Diabetes or Hyperglycemia								
Yes	4135	1583 (38.3)	1	-	-	1	-	-
No	28991	7714 (26.6)	0.584	0.546-0.626	<0.001	0.900	0.834-0.970	0.006
Hypertension								
Yes	13241	4533 (34.2)	1	-	-	1	-	-
No	19885	4764 (24.0)	0.605	0.577-0.635	<0.001	0.877	0.829-0.928	<0.001

* Country random effect: estimate = 0.363; standard error = 0.273; p = 0.184.

## RESULTS

From a total of 58,489 individuals that participated in wave 4 of the SHARE survey, we selected non-institutionalised individuals aged 50 years or older, who answered all of the questions included in this work. Accordingly, we included 33,580 individuals in this analysis. Participants had a mean age of 65.4 (sd = 10.0) years; 56.4% were female. Table I shows the entire sample set characterised by gender, country and number of education years. The standardised prevalence of impaired cognitive performance in tests of cognitive abilities are shown in Table II. The prevalence of impairment was of 28.02%, 27.89% and 20.75% for perceived memory, verbal fluency, and numeracy, respectively, in the 16 evaluated countries. The geographic distribution was heterogeneous between the evaluated countries (Table II). Regarding memory complaints, the highest prevalence was found in Estonia [46.18 (45.71-46.66%)] and Portugal [44.41 (43.94-44.88%)], and the lowest prevalence was found in Denmark [8.82 (8.62-9.03%)]. For numeracy impairment, the highest prevalence was found in Spain [46.02 (45.55-46.50%)] and Portugal [36.92 (36.50-37.35%)], and the lowest prevalence was found in Switzerland [9.04 (8.83-9.25%)]. For verbal fluency impairment, the highest prevalence was found in Portugal [63.11 (62.55-63.67%)] and Italy [62.57 (62.01-63.12%)], and the lowest prevalence was found in Denmark [6.48 (6.31-6.67)]. The prevalence of cognitive impairment found in participants from Germany, Sweden and Poland must be evaluated with caution due to the small number of individuals included in the analysis (Table I).

To evaluate variables associated with the cognitive abilities identified in this study, we excluded data from three countries (Germany, Sweden and Poland) from this analysis due to the small number of individuals that remained after exclusion of cases with missing values (less than 5%). The remaining 33,126 individuals included in this analysis had a mean age of 65.4 (SD = 10.0) years; 56.5% were female. Via analysis of all countries (except Germany, Sweden and Poland) using unadjusted models, we found a significant association among all studied explanatory variables with the three cognitive abilities, i.e. perceived memory (Table 3), verbal fluency (Table 4), and numeracy (Table 4). However, from adjusted models we found that fewer years of education, being a smoker or former smoker, having more than two chronic diseases, and having diabetes, hyperglycemia or a history of heart attack or stroke were independent variables associated with perceived memory, verbal fluency and numeracy impairment (Tables 3, 4 and 5). We also found independent associations between physical inactivity with verbal fluency and numeracy impairment, females had more complaints about perceived memory and poorer performance in numeracy, older participants had more complaints about perceived memory and poorer performance in verbal fluency, and participants with hypertension had more complaints about memory.

## DISCUSSION

In this work, a high and heterogeneous prevalence of impairment in cognitive function was found in 16 evaluated European countries. Southern European countries (Spain, Portugal, Italy and Slovenia) as well as Eastern Europe (Hungary, Poland, Czech Republic) showed the highest prevalence of cognitive impairment in the evaluated abilities. Conversely, Western European countries (Austria, Belgium, Switzerland, France, and the Netherlands) as well as Northern Europe (Denmark, Sweden) showed the lowest rates of cognitive impairment. These results are in accordance with previous studies that showed significant variation in cognitive performance across countries [15,16].

The findings suggest that older ages, being female, and having less education are associated with poorer cognitive performance. Not surprisingly, increased age is significantly related to poorer cognitive performance. Indeed, the influence of ageing on cognitive function has been extensively described, especially regarding processing speed, memory and language [17]. Evidence shows that cognitive decline during the ageing process corresponds with structural brain changes such as cerebral atrophy, ventricular enlargement and the loss of neuronal networks [18-20].

Education has also been shown to be strongly associated with cognitive function, with higher education levels (more than 12 years) having a protective effect by significantly decreasing the risk of developing cognitive impairment. A positive association between cognition and education in the elderly has been reported previously [21]. Data from the English Longitudinal Study on Ageing supports our results, showing that among English seniors, education increases memory in both genders as well as executive function in males. This result could explain some of the differences observed between countries, since participants from Central and Northern Europe with better cognitive function are also in regions with relatively high education levels, as has been reported previously [15,19]. Moreover, cognitive tests may reflect the quality of education as well as socioeconomic status, social interactions, labor force participation or cognitive reserve [22]. There is also evidence that improvements in mental performance are related to changes in brain structure, affecting synaptic density, hippocampal volume and cortical thickness [18].

**Table 5:** Association of explanatory variables with numeracy impairment; unadjusted and adjusted models.

	n	n (%) ≤15 correct words	Unadjusted model			Adjusted model *		
	33126		OR	CI 95	p	OR	CI 95	p
Female	18709	5285 (28.2)	1	-	-	-	-	-
Male	14417	3959 (27.5)	0.962	0.916-1.009	0.113	-	-	-
Age, years								
>75	6460	1898 (29.4)	1	-	-	1	-	-
65-74	9504	2617 (27.5)	0.913	0.852-0.980	0.011	0.937	0.871-1.008	0.082
55-64	12048	3392 (28.2)	0.942	0.881-1.007	0.078	0.974	0.908-1.046	0.467
50-54	5114	1337 (26.1)	0.851	0.784-0.924	<0.001	0.870	0.798-0.949	0.002
Education, years								
>12-y	9376	1280 (13.7)	1	-	-	1	-	-
≤12-y	23750	7964 (33.5)	3.191	2.991-3.405	<0.001	3.075	2.881-3.283	<0.001
BMI. Kg/m^2^								
≥30	7652	2162 (28.3)	1	-	-	-	-	-
25-29.9	13634	3827 (28.1)	0.955	0.895-1.020	0.172	-	-	-
18.5-24.9	11840	3255 (27.5)	0.928	0.867-0.993	0.031	-	-	-
Smoking								
Smoker	18156	5583 (30.8)	1	-	-	1	-	-
No	14970	3661 (24.5)	0.729	0.694-0.766	<0.001	0.718	0.683-0.755	<0.001
Physical inactivity								
No	29013	7997 (27.6)	1	-	-	1	-	-
yes	4113	1247 (30.3)	0.875	0.814-0.939	<0.001	0.906	0.839-0.977	0.011
Number of chronic diseases								
>2	9050	3063 (33.8)	1	-	-	1	-	-
≤2	24076	6181 (25.7)	0.675	0.641-0.711	<0.001	0.920	0.863-0.981	0.011
Comorbidities								
Heart attack								
Yes	4805	1717 (35.7)	1	-	-	1	-	-
No	28321	7527 (26.6)	0.651	0.610-0.694	<0.001	0.785	0.730-0.844	<0.001
Stroke								
Yes	1485	653 (44.0)	1	-	-	1	-	-
No	31641	8591 (27.2)	0.475	0.427-0.528	<0.001	0.574	0.513-0.642	<0.001
Diabetes or Hyperglycaemia								
Yes	4135	1542 (37.3)	1	-	-	1	-	-
No	28991	7702 (26.6)	0.608	0.568-0.651	<0.001	0.712	0.660-0.768	<0.001
Hypertension								
Yes	13241	4058 (30.6)	1	-	-	-	-	-
No	19885	5186 (26.1)	0.798	0.760-0.838	<0.001	-	-	-

* Country random effect: estimate = 0.178; standard error = 0.147; p = 0.226

Our analyses show that lower performance in perceived memory, verbal fluency, and numeracy were strongly related to comorbidities represented by more than two self-reported chronic diseases. This is in accordance with other studies suggesting that individuals with poor health status are at a higher risk for significant cognitive impairment or dementia [23,24]. Existing data support the notion that the number of chronic diseases could be a marker of cumulative exposure to vascular risk factors [8]. The underlying mechanism seems to be related to peripheral inflammatory markers, a potential indicator of global inflammatory processes that may increase the risk for dementia through their role in the development and progression of cardiovascular disease [25].

Cardiovascular risk factors, including smoking, physical inactivity, hypertension and a previous diagnosis of diabetes, hyperglycemia, stroke or heart attack were independent variables associated with cognitive impairment. Stroke is the major risk factor for impaired cognitive function in numeracy, perceived memory and verbal fluency. A history of strokes almost doubled the risk, which agrees with other studies [26]. Although the underlying process of brain damage requires more research, it is known that vascular dysfunction and endothelial injury are important mediators of atherosclerosis development, damage to medium-large arteries, and probably to smaller brain arteries as well [27]. Studies investigating asymptomatic or “silent” strokes suggest that the development of AD may be positively associated with underlying vascular problems [28]. These findings suggest that strokes play a role in the etiology of dementia, which is supported by a recent meta-analysis that demonstrated a positive association between the risk for AD and stroke [29]. Growing evidence has identified cardiovascular risk factors, such as diabetes and hypertension, as predictors of cognitive decline post-stroke, especially after the occurrence of a second stroke [8]. High cardiovascular risk patients may be a target subpopulation in which to improve screening to prevent another cardiovascular event or cognitive decline [3].

Coronary heart disease (“heart attack”) increased the risk of having lower cognitive performance in all three cognitive domains evaluated, which is in accordance with recent research that identified an association between cognitive decline and small, clinically silent infarcts and coronary artery disease [30]. In the Women's Health Initiative Memory Study [31], in women over 65 years old with a history of infarction or invasive vascular procedures, the risk of cognitive decline was double that of the subgroup without myocardial infarction. Moreover, this study suggested that cognitive impairment was positively associated with heart and aortic atherosclerosis, particularly in patients with angina pectoris, carotid endarterectomy, peripheral vascular disease, and coronary bypass surgery [31]. However, the relationship between coronary artery disease and cognitive impairment or AD remains unclear, with conflicting reports [32,33].

Diabetes or hyperglycemia was independently associated with impaired cognitive performance in the three cognitive domains: numeracy, perceived memory and verbal fluency. This is in line with recent research that supports a positive association between type 2 diabetes and a higher incidence of cognitive decline [34]. Potential mechanisms involved in the pathogenesis of cognitive dysfunction and neurodegeneration include acute and chronic hyperglycemia and insulin resistance [35,36]. Although secreted peripherally, insulin also plays a particular role in cognitive function, learning, and memory [37]. However, more studies are needed to clarify the association between high glucose levels and dementia. Hypertension has been recognised as an important risk factor for cognitive decline and mild cognitive impairment [38]. It is well-established that hypertension pressure can increase the risk of stroke and heart disease and decrease life expectancy. Many studies have associated the decline in cognitive function with hypertension in the elderly [39-41]. High blood pressure during middle age has been associated with future cognitive decline and dementia, including AD. Although the mechanisms underlying hypertension and cognitive dysfunction are not yet well understood, cerebral vessel disorder, which is pulsatile pressure changes in the cerebral microvasculature and possibly increased production of amyloid-beta, seems to be involved [42]. Although increased evidence suggests a contribution of hypertension to cognitive decline during middle age, data on its role later in life is not consistent. Some studies reported a significant positive effect of hypertension on cognitive decline [43] while others found a U-shaped relationship between hypertension and cognitive performance [44].

The increased risk of cognitive decline and dementia in smokers compared to non-smokers is supported by several prospective studies. These observations were corroborated by the Honolulu-Asia Aging Study in which a dose-response relationship was established between the number of pack-years and the deposition of amyloid substance in the brain [45]. It has been widely recognised that tobacco has important effects on the cardiovascular system, including the development of atherosclerosis and small vessel brain disease [8]. In addition to the direct neuronal damage caused by neurotoxins, data from the Whitehall II study showed a selective risk for certain cognitive domains [46], possibly because nicotine has psychoactive effects and stimulates acetylcholine receptors in the brain [8].

There is a wealth of literature on the association between physical activity and improved cognitive performance. Numerous underlying hypotheses have tried to explain how physical activity might protect brain health. The prominent theories are that physical activity leads to increased cerebral blood flow, neurogenesis, angiogenesis, and synaptic plasticity while at the same time reducing inflammation and improving resistance to brain insults [47-49].

Some limitations of this study require discussion. Firstly, the impact of other variables that might influence cognitive performance was not evaluated. This is a cross-sectional study while the associations being measured happened over a long period, which could be a limitation of this study. Additionally, even though this work is cross-sectional, SHARE data collection is ongoing and future observations over an extended period will allow for analyses between cognitive decline and its associated cardiovascular risk factors. The sampling procedure could also influence some of the results. In Northern and Western European countries, there is a higher proportion of the elderly population that is institutionalised, which could have excluded an unhealthier population from our sample. The sample from Southern Europe (Spain, Portugal and Italy), where old people often live with their families, has a higher chance of being included in the SHARE survey. Moreover, some variables presented high non-response ratios, which might affect the results. Finally, there are some contradictory studies on self-reported measures, a practice criticised by some authors but considered reliable by others. To the best of our knowledge, this is the first study to directly compare elderly populations from 16 European countries and provides evidence that cardiovascular risk factors are strongly associated with impaired cognitive function, even after accounting for sociodemographic and health status. Moreover, we report important results about the heterogeneity across Europe, with valuable policy implications on late life cognitive decline prevention that could be implemented in the clinical setting.

In conclusion, impaired cognitive function in terms of verbal fluency, numeracy and perceived memory is highly prevalent but unequally distributed across Europe. This is associated with multiple factors, namely cardiovascular risk factors, most of which can be prevented or treated.
